# Hidden structural and chemical order controls lithium transport in cation-disordered oxides for rechargeable batteries

**DOI:** 10.1038/s41467-019-08490-w

**Published:** 2019-02-05

**Authors:** Huiwen Ji, Alexander Urban, Daniil A. Kitchaev, Deok-Hwang Kwon, Nongnuch Artrith, Colin Ophus, Wenxuan Huang, Zijian Cai, Tan Shi, Jae Chul Kim, Haegyeom Kim, Gerbrand Ceder

**Affiliations:** 1 Department of Materials Science and Engineering, University of California Berkeley, Berkeley, CA 94720 USA; 2 Materials Sciences Division, Lawrence Berkeley National Laboratory, Berkeley, CA 94720 USA; 3 School of Chemistry, University of St Andrews, St Andrews, KY16 9ST UK; 4 Department of Materials Science and Engineering, Massachusetts Institute of Technology, Cambridge, MA 02139 USA; 5 National Center for Electron Microscopy, Molecular Foundry, Lawrence Berkeley National Laboratory, Berkeley, CA 94720 USA; 6 Department of Chemical Engineering and Materials Science, Stevens Institute of Technology, Hoboken, NJ 07030 USA

## Abstract

Structure plays a vital role in determining materials properties. In lithium ion cathode materials, the crystal structure defines the dimensionality and connectivity of interstitial sites, thus determining lithium ion diffusion kinetics. In most conventional cathode materials that are well-ordered, the average structure as seen in diffraction dictates the lithium ion diffusion pathways. Here, we show that this is not the case in a class of recently discovered high-capacity lithium-excess rocksalts. An average structure picture is no longer satisfactory to understand the performance of such disordered materials. Cation short-range order, hidden in diffraction, is not only ubiquitous in these long-range disordered materials, but fully controls the local and macroscopic environments for lithium ion transport. Our discovery identifies a crucial property that has previously been overlooked and provides guidelines for designing and engineering cation-disordered cathode materials.

## Abstract (web summary)

The average crystal structure largely governs the Li diffusion kinetics in well-ordered cathode materials. Here the authors show this rule does not hold true for cation-disordered analogues. Cation short-range order is not only ubiquitous but also controls the Li transport behavior.

## Introduction

The development of cost-effective lithium (Li) ion batteries depends on the discovery of high-energy-density cathode materials composed of non-precious elements^1^. Rational design of cathodes requires an understanding of the precise role that each chemical component has in determining performance. Traditionally, one thinks of redox-active elements, such as Ni and Co, and stabilizers, such as Mn^4+^ in NMC-class materials^2^. However, we demonstrate in this paper that in the recently discovered class of Li-excess cation-disordered rocksalt (DRX) cathodes, the chemistry of then non-redox-active stabilizers plays a critical role in performance through subtle structural changes.

DRX materials were recently shown to have facile Li transport enabled by a percolating network of Li-rich environments^3^. Their ability to function without requiring cation ordering has enabled novel cathodes with remarkable chemical diversity^3,4^. Many new cathode materials, in some cases containing only earth-abundant elements (e.g., Fe, Mn, and Ti) have been developed in this category, such as Li_1.3_Mn_0.4_Nb_0.3_O_2_^5^, Li_1.2_Mn_0.4_Ti_0.4_O_2_^6^, Li_1.2_Ni_1/3_Ti_1/3_Mo_2/15_O_2_^7^, Li_4_Mn_2_O_5_^8^, and Li_2_FeV_0.5_Ti_0.5_O_4_^9^ as well as their fluorinated variants^10–14^. A prevailing assumption when studying DRX cathodes is that all the cation species are randomly distributed.

**Fig. 1:**
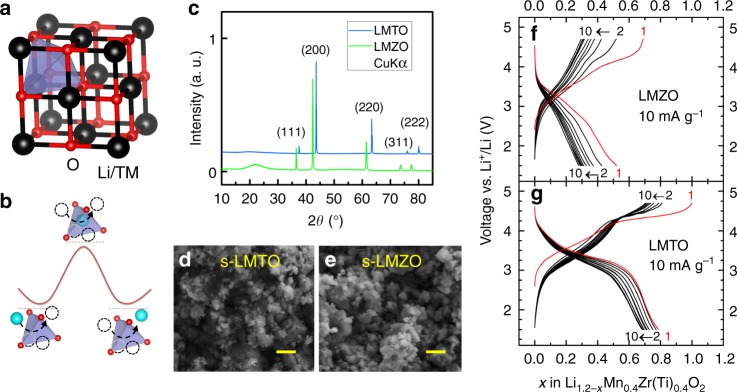
Characterization and electrochemical performance of Li_1.2_Mn_0.4_Zr_0.4_O_2_ (LMZO) and Li_1.2_Mn_0.4_Ti_0.4_O_2_ (LMTO). **a** Crystal structure of an ideal cation-disordered rocksalt-type lithium metal oxide. The black spheres represent metal cations (including lithium and TMs), and the red spheres represent oxygen anions. Both cations and anions are in octahedral coordination. The highlighted blue tetrahedral site represents a typical migration pathway for Li diffusion. **b** Schematic energy landscape of Li migration from its octahedral coordination through a tetrahedral vacancy into another octahedron. The energy barrier depends on the local environment and size of the tetrahedron. The migrating Li ion is highlighted in cyan. **c** XRD patterns of LMZO and LMTO indexed according to the rocksalt structure. The low-angle shift in the pattern of LMZO compared with that of LMTO indicates the larger lattice parameter of LMZO. **d**, **e** SEM images (scale bars, 500 nm) of shaker-milled LMTO (s-LMTO) and LMZO (s-LMZO) with similar particle sizes of ~100 nm. **f**, **g** Voltage profiles of LMZO and LMTO between 1.5 and 4.7 V for the first 10 cycles at room temperature

Figure 1a presents a typical DRX crystal structure. The highlighted tetrahedron represents a channel through which Li migrates^15^ (Fig. 1b). The Li-migration barrier depends on the tetrahedron height^3,16–18^ and the number of transition metal (TM) ions within the environment (i.e. 0-TM, 1-TM, or 2-TM)^4^. If the cation arrangement is random, any DRX with the same Li to TM ratio should have an equivalent distribution of Li-migration channel types and thus similar Li transport properties.

However, in this study, we show that even minor deviations from randomness, not detectable by typical X-ray diffraction (XRD), can have profound influence on performance. We find that cation short-range order (SRO) is important in determining the amount of kinetically extractable Li in DRX materials as the Li transport relies on percolation through a 3D network. We compare two Li-excess DRXs, Li_1.2_Mn_0.4_Ti_0.4_O_2_ (LMTO) and Li_1.2_Mn_0.4_Zr_0.4_O_2_ (LMZO). Based on their chemical similarity, these materials would be expected to have comparable electrochemical properties, as Zr^4+^ and Ti^4+^ are isoelectronic and their sole role is to charge compensate for the excess Li. If anything, the larger ionic radius of Zr^4+^ should result in a larger lattice parameter for LMZO, which is generally considered beneficial for Li mobility^16–18^. However, contrary to these expectations, we observe that the performance of LMTO is considerably better than that of LMZO. We reveal through a combination of electron diffraction, neutron pair distribution function measurements, and cluster-expansion Monte Carlo simulation that the difference in the performance of LMTO and LMZO is due to different cation SRO, which controls the population and connectivity of Li-migration channels. We further identify general rules that govern the relationship between SRO and Li transport by expanding our analysis to other combinations of TMs. These results indicate the importance of SRO and provide another important handle to tailor the performance of DRX cathode materials, in addition to the already large compositional flexibility.

## Results

### Synthesis and electrochemistry

We synthesized LMTO and LMZO using a solid-state method and verified the compositions of the products to be nearly identical to the target compositions using elemental analysis (Supplementary Table 1 and Supplementary Note 1). XRD patterns (Fig. 1c) reveal a DRX structure. Rietveld refinement indicates that the lattice parameter of LMZO (*a* = 4.27 Å) is larger than that of LMTO (*a* = 4.15 Å), as expected (Supplementary Figure 1). Scanning electron microscopy (SEM) images of shaker-milled LMTO and LMZO (Fig. 1d and e) confirm particle sizes of ~100 nm for both materials. The galvanostatic voltage profiles of LMZO and LMTO are presented in Fig. 1f and g. Consistent with a previous report by Yabuuchi et al.^6^, LMTO delivers a large first-cycle capacity of ~260 mAh g^−1^ at room temperature, which corresponds to 0.79 Li per formula unit (*f.u*.). However, LMZO delivers a much smaller discharge capacity (0.52 Li per *f.u*.) (ex situ XRD patterns and supplementary electrochemical tests are shown in Supplementary Figures 2, 3 and described in Supplementary Note 2). In addition, the average discharge voltage for the first cycle is lower in LMZO (2.75 V) than in LMTO (3.08 V).

**Fig. 2:**
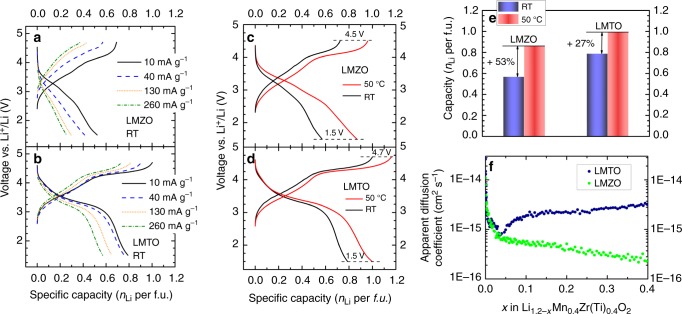
Rate capability tests, comparison between high-temperature and room-temperature galvanostatic cycling of LMTO and LMZO, and PITT measurements. First-cycle voltage profiles of LMZO (**a**) and LMTO (**b**) when cycled between 1.5 and 4.7 V at 10, 40, 130, and 260 mA g^−1^. First-cycle galvanostatic voltage profiles of **c** LMZO and **d** LMTO at 50 °C and room temperature at 10 mA g^−1^. **e** First-cycle reversible capacities of LMZO and LMTO at high temperature and room temperature. **f** Li chemical diffusion coefficients of LMZO (green) and LMTO (blue) obtained from fitting the room-temperature PITT data at various Li contents

The Li diffusion is a key factor in determining the observed capacity^19,20^, especially in LMTO and LMZO. The computed voltage profiles obtained by enumerating a large number of possible configurations (Supplementary Figure 4 and Supplementary Note 3) indicate that both materials should cycle in a comparable voltage window and their capacity would not be limited by thermodynamics. Indeed, the rate capability of LMTO (Fig. 2b) is clearly better than that of LMZO (Fig. 2a). To further test whether the capacity of LMZO is limited by kinetics, galvanostatic cycling at both room-temperature and 50 °C was performed, as shown in Fig. 2c, d. The upper cutoff voltage for LMZO is set at 4.5 V, while that of LMTO is set at 4.7 V. This is because we find that cycling LMZO to 4.7 V at 50 °C provides little extra reversible capacity compared to a 4.5 V-cutoff and causes a side reaction above 4.5 V which seems to lead to increased polarization and subsequent discharge voltage fade. The difference between room-temperature and 50 °C indicates pronounced kinetic limitations in LMZO over the entire voltage range, unlike LMTO, which only shows improvement at 50 °C near the upper cutoff voltage. As shown in Fig. 2e, at 50 °C, LMZO delivers a reversible capacity of 0.87 Li per *f.u*., a 53% increase from that at room temperature. In contrast, the capacity of LMTO improves by only 27% to 1.0 Li per *f.u*., when cycled at 50 °C. The Li chemical diffusivities (*D*_Li_) of LMTO and LMZO were determined using the potentiostatic intermittent titration technique (PITT)^21–23^ during the initial charge from open-circuit voltages to 4.7 V (Fig. 2f, Supplementary Figure 5 and Supplementary Note 4). It should be noted that the apparent Li diffusivity deduced from PITT includes the contribution from bulk and grain boundaries whereas the migration barriers from nudged elastic band calculations would only impact the bulk diffusivity. Besides, given that the chemical diffusivity measured by PITT is a product of the thermodynamic factor and the self-diffusivity, the apparent diffusivities (*D*_Li_’s) of the two materials at the beginning of charge (*x* < 0.05) are dominated by the large thermodynamic factors originating from the steep voltage increase in the region and are therefore not a representation of the intrinsic Li mobility. We observe that the Li diffusivity in LMTO is much higher than that in LMZO, confirming that the capacity of LMZO is more limited by Li transport kinetics. Given the validity of the PITT method in solid-solution systems, we plot the region from *x* = 0 to *x* = 0.4 where Mn^3+/4+^ oxidation and a solid-solution reaction dominate^6,24^.

### Local-structure characterization

**Fig. 3:**
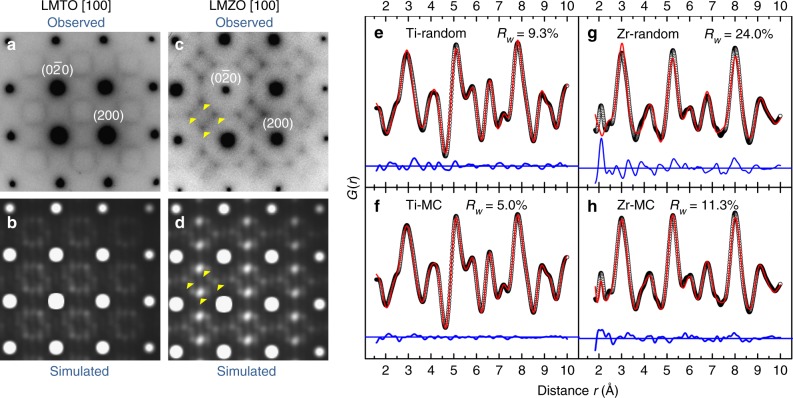
Experimental observation and computational simulation of short-range order (SRO) in LMTO and LMZO. ED patterns of LMTO (**a**) and LMZO (**c**) along the zone axis [100]. The round spots are indexed to the F*m–*3*m* space group, while the diffuse scattering patterns nearby are attributed to SRO. Several intensity maxima in the diffuse scattering patterns are highlighted with yellow arrows in LMZO. Simulation of ED patterns for LMTO (**b**) and LMZO (**d**) along the same zone axis shows good agreement with experimental observation. Refinement of NPDF data of LMTO (**e**, **f**) and LMZO (**g**, **h**) using the random model (**e**, **g**) and MC-equilibrated structural models (**f**, **h**). The experimental data are plotted as black open circles. The calculated values are plotted as solid red lines. The difference between observation and calculation is plotted as solid blue lines

The discovery that two almost identical materials exhibit significantly different Li transport led us to investigate the subtle structural differences between LMTO and LMZO. Figure 3a, c and Supplementary Figure 6 present the electron diffraction (ED) patterns of LMTO and LMZO. Aside from the reflection spots that can be indexed with a DRX structure, we also observe diffuse scattering patterns surrounding the Bragg reflections, suggesting the existence of SRO. The formation of SRO can be understood as a preferred local arrangement of species, resulting in non-vanishing patterns in reciprocal space^25–28^. Notably, the diffuse scattering patterns are completely different, both in shape and orientation, for the two materials, indicating significant difference in SRO. Based on previous characterization of SRO in oxides and alloys, the SRO patterns in LMTO is characteristic of octahedral cation clusters similar to the [Li_3_Fe_3_] in cubic-LiFeO_2_^29^, whereas that in LMZO is likely associated with tetrahedral cation clusters^30^. In addition, the intensity of the diffuse scattering pattern of LMZO is noticeably stronger at several maxima highlighted with yellow arrows, suggesting more pronounced SRO in LMZO than in LMTO. These intensity maxima at $\left\langle {1\frac{1}{2}0} \right\rangle$ positions are among the four classes of special points in an FCC lattice where more than two symmetry elements intersect and do not correspond to any long-range order^31^. These characteristic features observed in the diffuse scattering patterns are well reproduced by simulation (Fig. 3b, d) based on thermodynamically representative structures, which we obtain from Monte Carlo (MC) sampling at 1000 °C with a cluster expansion (CE) Hamiltonian parameterized to fit the rocksalt configurational energies derived from density-functional theory (DFT)^32,33^. Detailed analysis of the model structures is presented in the next section.

Neutron pair distribution function (NPDF) measurements were performed to precisely characterize the SRO in LMTO and LMZO. In NPDF analysis, Fourier transformation of the total scattering data to real space is performed, thereby providing additional information about SRO that is hidden in diffraction patterns^34^. The refinement of the NPDF patterns of LMTO and LMZO is presented in Fig. 3e–h. We use two structural models for the refinement: One is a random model that assumes a random cation distribution in a distortion-free lattice, and the other is the MC-derived structural model described above. The random model produces a reasonable fit for LMTO but not for LMZO, as indicated by the goodness-of-fit values (*R*_*w*_) (Fig. 3e, g, Supplementary Figure 9, Supplementary Note 5 and Supplementary Table 2). In LMZO, the simulation based on the random model differs significantly from the experimental observation near 2, 3, 5, and 8 Å (Fig. 3g). Nevertheless, in the longer range, the random model produces a good fit for both materials (Supplementary Figure 7 and Supplementary Table 3). These results suggest that LMZO has significantly more SRO than LMTO. As a comparison, the refinement using the MC configurations, presented in Fig. 3f, h, shows significant improvement for both compounds (details in Supplementary Figure 8, Supplementary Table 4 and Supplementary Method 1).

Combining the analysis of ED and NPDF, we find that LMTO and LMZO differ in their SRO and that the ab initio MC structures simulated near the synthesis temperature are precise manifestations of the SRO in these materials.

### Modeling of Li transport environments in LMTO and LMZO

The structures obtained from MC simulation uncover the atomistic nature of the SRO, enabling further analysis of the local and macroscopic Li-transport environments in LMTO and LMZO.

**Fig. 4:**
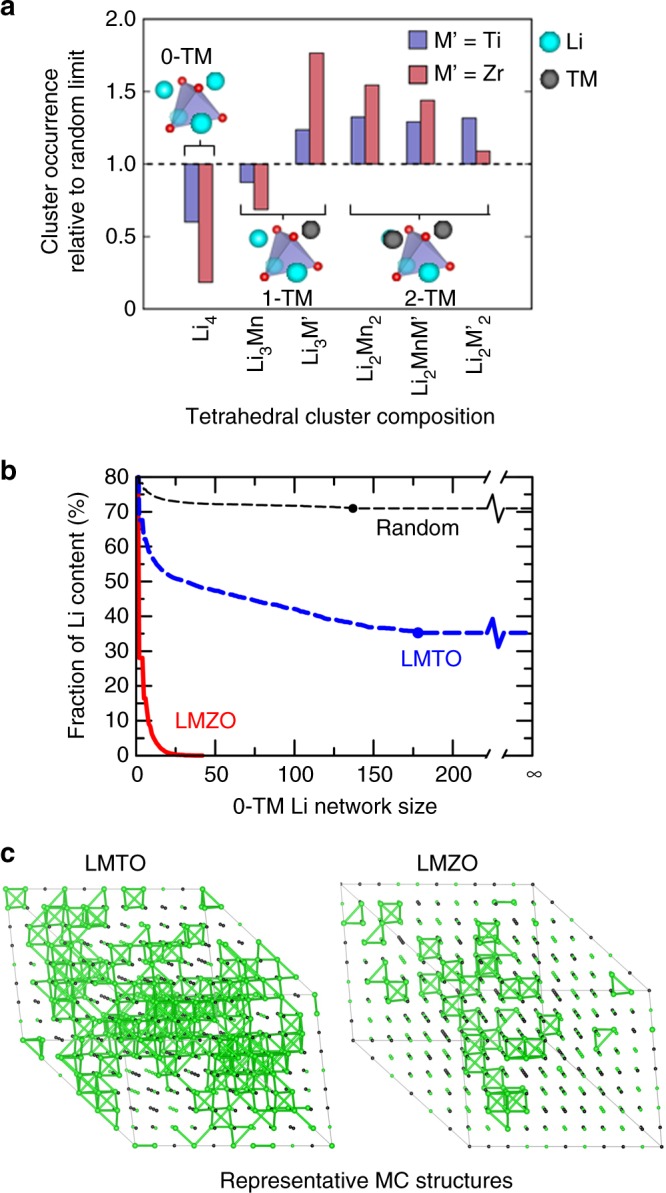
Local cation cluster and macroscopic Li connectivity analysis based on MC-derived structures for LMTO and LMZO at 1000 °C. Each MC structure contains 480 cation sites, of which 288 are decorated with Li ions. **a** Occurrence of various tetrahedral clusters (0-TM, 1-TM, 2-TM) in LMTO (blue) and LMZO (red) as compared to the random limit. **b** Connectivity plots of LMZO and LMTO showing the fraction of Li content in networks of at least a certain number of Li sites. A Li network is defined as all the Li sites that are inter-connected through 0-TM channels. Each plot is averaged over 600 sampled MC structures. The result for a random cation distribution with the same Li to TM ratio is also plotted as a reference. The plots are truncated at the percolating Li contents (marked by dots) and extended to infinity for LMTO and the random case. LMZO is not percolating. **c** Representative MC structures for LMTO and LMZO. Li ions are labeled with green spheres and 0-TM connected Li sites are bridged with green bonds

In DRX materials, local environments can be characterized by the occurrence of cation clusters, among which the ones most relevant to Li transport are tetrahedral clusters, i.e., Li-migration channels^19^. Because of their connectivity in the structure, their population is not completely independent, a phenomenon that has been recognized early on when studying the entropy of FCC systems^35^. Figure 4a summarizes the occurrence of various tetrahedral clusters in LMTO and LMZO relative to a random case. We observe that the occurrence of Li_4_ tetrahedra (i.e. 0-TM channels), which is the most important for good Li transport, is significantly lower in LMZO than in LMTO, although both materials have lower Li_4_ population than for a random cation distribution. Conversely, the population of Li_3_M tetrahedra, i.e. 1-TM channels, is much higher in LMZO than in LMTO. More specifically, the Li_3_Zr clusters account for 31% of all cation tetrahedra in LMZO, as compared to 22% for Li_3_Ti in LMTO and 17% for Li_3_M’ in the random case (Supplementary Table 5). Such a high population of 1-TM channels in LMZO is detrimental for Li transport as it was previously demonstrated that in a typical DRX, the migration barrier through a 1-TM channel is on average 200 meV higher than that through a 0-TM channel^3^.

These observations indicate that SRO strongly modifies the population of local tetrahedral clusters: LMTO favors Li_4_ clusters more than LMZO does; while LMZO contains more Li_3_M (especially Li_3_Zr) clusters.

While the population of Li_4_ tetrahedra is critical for local Li migration, a sufficient connectivity between these environments is another key criterion to ensure macroscopic Li transport. Figure 4b presents a connectivity analysis for LMTO and LMZO as compared to a random cation distribution. A connectivity plot, averaged over 600 sampled MC structures, shows the fraction of Li content in Li networks of more than a certain number of 0-TM inter-connected Li sites (Supplementary Method 2). The fraction of Li content initially decreases with an increasing network size and finally plateaus at the percolating Li level, which is considered the lower bound of accessible Li. Figure 4b suggests that LMTO has more extensively connected 0-TM Li networks compared to LMZO, although both materials show worse Li connectivity than the random case. Specifically, in LMTO, nearly 40% of Li is in 0-TM networks of more than 100 Li sites and ~35% of Li is percolating. In contrast, in LMZO, the fraction of Li in networks of more than 25 Li sites is already vanishingly small and the material is not percolating.

To visualize the Li diffusion pathways in LMTO and LMZO, representative MC structures are shown in Fig. 4c. The 0-TM connected Li networks are highlighted in green. From these images, it is clear that LMTO has extensive 0-TM Li networks that are well connected and should allow facile Li transport, whereas LMZO lacks 0-TM channels, thereby impeding Li diffusion.

We may draw some useful insight about the impact of SRO on the preferred cation configurations around tetrahedral sites based on the relationship between SRO and its corresponding ground states. This is because SRO is generally considered an “intermediate state” between a random state and an ordered low-temperature ground state, and therefore, it often resembles the low-temperature long-range order^30^. In DRX oxides with multiple TM species, our calculations indicate that phase separation into ground-state compounds is preferred at low temperatures. For example, LMZO is thermodynamically favored to decompose into LiMnO_2_ and Li_2_ZrO_3_ at low temperatures according to our calculations. Li_2_ZrO_3_ is known to have a *γ*-LiFeO_2_-like structure^36^, in which most cation tetrahedra are made up out of 2Li and 2Fe ions, which gives the compound very poor Li percolation^4^. Indeed, not even one Li site in Li_2_ZrO_3_ is connected to other Li sites through 0-TM channels even at a Li-excess level of 33.3%. In contrast, Li_2_TiO_3_ adopts a Li_2_MnO_3_-like structure^37^, which contains a large amount of tetrahedra with 1TM and 3Li as well as tetrahedra with 4Li, which makes every Li site connected with other Li sites. The SRO and percolation properties observed in the cation-disordered materials are reminiscent of the low-temperature ground states, which explain why LMTO favors the segregation of Li in tetrahedra while LMZO exhibits poor Li conductivity.

### Chemistry dependence of SRO and Li transport environments

**Fig. 5:**
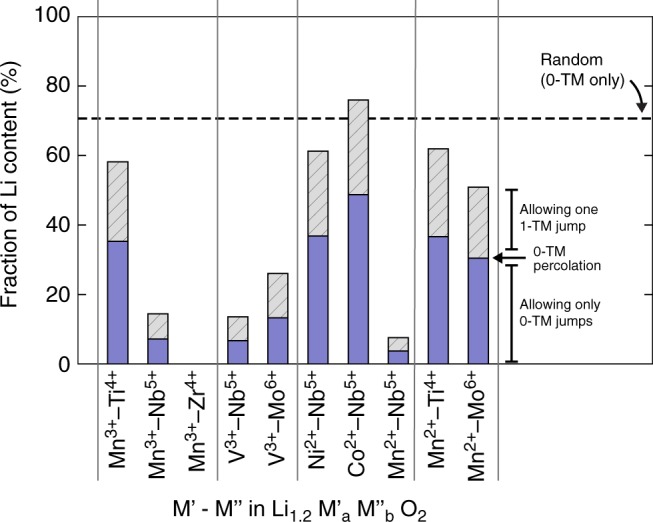
Fraction of Li content made accessible by the percolating network in a specific DRX Li_1.2_M’_*a*_M”_*b*_O_2_. Two conditions are considered: allowing only 0-TM jumps (blue), or allowing any given Li to make a single 1-TM jump before reaching the 0-TM percolating network (gray). A percolating Li level is considered the lower bound of accessible Li. The dotted line marks the fraction of Li accessible within the 0-TM percolating network in the random structure limit. The stoichiometry of each Li_1.2_M’_*a*_M”_*b*_O_2_ compound is constructed such that charge neutrality is retained. The various combinations of TM species M’–M” are indicated along the *x*-axis

With the understanding of how SRO affects Li transport in LMZO and LMTO, we can investigate other combinations of TMs to determine how the SRO-affected Li transport environments vary with chemistry. Figure 5 shows the accessible Li contents based on the percolation theory^3^ in a variety of Li_1.2_M’_*a*_M”_*b*_O_2_ compounds under two conditions: (i) allowing only 0-TM jumps or (ii) allowing any given Li to make a single 1-TM jump before reaching the 0-TM percolating network. The rationale behind the chosen conditions is that although sufficient 0-TM channels are required for macroscopic Li transport, in reality, on the atomic scale Li ions can occasionally overcome higher migration barriers through 1-TM channels on the time scale of battery charge and discharge. Besides, since *d*^0^ TMs are known to promote cation disorder in Li-TM oxides^38^, all the hypothetical compositions contain a redox-active TM as well as a *d*^0^ TM.

Consistent with the connectivity analysis, LMTO has a high accessible Li content of 35% with only 0-TM jumps, which increases to nearly 58% by allowing 1-TM jumps, whereas LMZO is not percolating under either condition. The accessible Li contents for Mn^3+^–Nb^5+^ are worse than LMTO, but still significantly better than those of LMZO, consistent with the good performance of the Mn^3+^–Nb^5+^ materials at elevated temperatures^5^. V^3+^–Nb^5+^ is quite similar to Mn^3+^–Nb^5+^, which might explain the limited first-cycle capacity of a previously reported Li-V-Nb-O DRX, which continuously increases upon cycling as the structure gets more disordered with V migration^39^. Overall, in all cases, compounds containing Ti^4+^ and Mo^6+^ lead to higher accessible Li contents than those containing Nb^5+^. Moving to divalent TM ions such as Ni^2+^ and Co^2+^, we find that they generally have higher accessible Li contents than the trivalent analogues, except that Mn^2+^–Nb^5+^ appears to be a poor-performing exception.

## Discussion

We have shown that SRO is critical in controlling Li-conductive environments and has a general dependence on chemistry. The remaining questions are thus what the microscopic origin of these trends is and how we can predict and manipulate SRO for the benefit of Li transport. Although the use of MC simulation is necessary to precisely reproduce SRO, empirical rules can be derived for intuitive prediction.

We find that the charge and size effects, which determine the stability of solid-state materials, also explain the trends in SRO. On the one hand, the high-valent TMs (e.g., Mn^3+^, Ti^4+^, Nb^5+^) in DRXs tend to repel each other and intimately mix with Li^+^ in order to keep local electroneutrality, thereby inhibiting Li segregation into Li_4_ tetrahedra. This charge effect becomes more pronounced with increasing metal valence, based on nearest-neighbor pair analysis (Supplementary Figure 10 and Supplementary Method 3). On the other hand, the size mismatch between high-valent TMs and Li^+^ facilitates Li segregation in order to minimize strain, counteracting the charge effect. The competition between the two effects is best demonstrated in the case of LMTO and LMZO, where Zr^4+^ exhibits a stronger net attraction to Li^+^ than Ti^4+^ despite their common valence (Supplementary Figure 10). One explanation for this phenomenon is that Ti^4+^ (0.605 Å) is much smaller than Li^+^ (0.76 Å), and therefore, the size effect tends to segregate Li^+^ from Ti^4+^. In contrast, the size of Zr^4+^ (0.72 Å) is close to that of Li^+^, meaning that electrostatics dominates the size effect, favoring a maximal separation between the high-valent Zr^4+^ and a corresponding local ordering between Zr^4+^ and Li^+^.

**Fig. 6:**
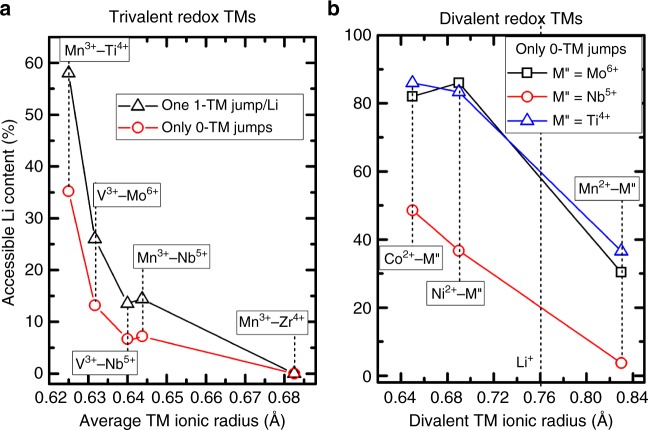
Correlation between accessible Li contents and ionic radii in various DRXs. **a** Accessible Li content as a function of the average TM ionic radius in DRXs composed of trivalent redox-active TMs, by allowing only 0-TM jumps (red) or allowing any given Li to make a single 1-TM jump before reaching the 0-TM percolating network (black). **b** Accessible Li content as a function of the divalent TM ionic radius in DRXs with divalent redox-active TMs and various stabilizers, by allowing only 0-TM jumps. The ionic radius of Li^+^ is marked at 0.76 Å. All the compositions listed have the same Li-excess level of 20%

However, the prediction of SRO becomes elusive when comparing DRXs with non-isoelectronic TMs, e.g., Li_1.2_Mn_0.4_Zr_0.4_O_2_ and Li_1.2_Mn_0.6_Nb_0.2_O_2_. In this case, there is a more complex tradeoff between interaction strengths and TM concentration in determining the degree of local ordering and Li segregation. Figure 6a shows a more intuitive relationship between accessible Li and chemistry, for DRXs composed of trivalent redox-active TMs. We observe a consistent negative correlation between the accessible Li content and the average TM ionic radius. The rationale behind the chosen *x-*axis is that in these DRXs, with a fixed Li-excess level, the average valence of the TMs is fixed accordingly and so is the electrostatic interaction strength between Li and TMs. Consequently, the size effect becomes dominant. Based on this correlation, we can further predict that high-valent metal species with large ionic sizes, e.g., Sc^3+^ (0.745 Å) and In^3+^ (0.80 Å), are likely to mix with Li^+^ and impede Li diffusion, whereas others with small sizes, e.g., Ga^3+^ (0.62 Å) and Sb^5+^ (0.60 Å), are likely to facilitate local Li segregation and efficient transport.

DRXs containing divalent TMs form another unique class because divalent ions have the proper average metal valence in a DRX oxide and therefore do not require mixing with Li^+^ to keep local electroneutrality. Additionally, they do not repel high-valent metals as much as trivalent ions do (Supplementary Figure 10), but can in turn mix with the high-valent metals to buffer their attraction to Li^+^. This buffer effect explains why DRXs containing divalent TMs generally have better Li_4_ segregation (Supplementary Figure 11) and higher accessible Li contents (Fig. 5) than their trivalent analogues. Figure 6b shows the accessible Li contents of DRXs containing divalent redox-active TMs and various stabilizers. We find that compounds containing Ti^4+^ and Mo^6+^ generally have higher accessible Li contents than the ones containing Nb^5+^, a phenomenon possibly explained by the tradeoff between the electrostatic interaction strength and metal concentration. In addition, within each plot, we observe a consistent dependence on the divalent-TM radius. A feasible mechanism would be that as the ionic radius of the divalent TM increases, the increasing size mismatch tends to segregate the divalent TMs from the high-valent TMs, weakening the buffer effect, which eventually vanishes for Mn^2+^, because its ionic radius is even larger than that of Li^+^. From Fig. 6b, we also notice that some of the metal pairs with the highest predicted percolating Li levels, i.e. Ni^2+^–Ti^4+^ and Ni^2+^–Mo^6+^, have been experimentally realized in a compound Li_1.2_Ni_1/3_Ti_1/3_Mo_2/15_O_2_ to deliver a high reversible capacity of 250 mAh g^−1^ when cycled at 10 mA g^–1^ between 1.5 and 4.5 V at room temperature^7^. However, it is worth noting that favorable cation SRO and a high kinetically accessible Li content only serve as necessary conditions and yet are insufficient to guarantee good capacity, which relies on various other factors, including the TM capacity, electronic conductivity, particle size, etc. For instance, although we predict a high kinetically accessible Li content >80% for Li_1.2_Ni_0.2_Ti_0.6_O_2_ (Fig. 6b), this compound is unlikely to deliver a high reversible capacity given its limited Ni content and the resulting small theoretical TM capacity.

Overall, we find that SRO controls the Li transport in DRXs by altering the distribution of 0-TM, 1-TM, and 2-TM channels as well as the connectivity between them. This observation is in contrast to stoichiometric layered oxides (e.g., LiNi_0.5_Mn_0.5_O_2_^40^ and Li[Ni_*x*_Mn_*x*_Co_1-2*x*_]O_2_^41,42^) where Li and TMs are well separated and any SRO in the TM layer imposes minimal impact on overall Li transport kinetics. We have also identified a few guidelines for the manipulation of SRO. (i) For DRXs containing high-valent metals, the average TM ionic radius is an important metric to measure the degree of Li segregation. The use of large metal ions such as Zr^4+^, Sc^3+^, and In^3+^ should be minimized. (ii) DRXs containing divalent TMs often facilitate Li segregation compared to their trivalent analogues due to the buffer effect of divalent TMs. This effect weakens as the ionic radius of the divalent TM increases. Therefore, small divalent TMs such as Co^2+^, Ni^2+^ are more favorable than Mn^2+^ and possibly Fe^2+^, Cu^2+^, Zn^2+^. These guidelines are contrary to the common intuition that larger metal ions expand the lattice and are therefore favorable for Li transport. Furthermore, given that SRO differs significantly with compositions and fully controls the local environment around both Li and O ions, we expect that voltage profiles as well as the conditions for oxygen redox will also be modified by SRO. We thus propose that the manipulation of SRO can also be relevant for the design and engineering of voltage profiles and oxygen redox, thereby enabling future optimization of this class of new cathode materials with unprecedentedly high capacities and compositional flexibility. It should also be noted that TM rearrangement may occur upon prolonged cycling, which might modify the nature of SRO somewhat during subsequent cycles.

In conclusion, motivated by an experimental puzzle where two extremely similar compounds exhibit different electrochemical performance, we prove that cation short-range order (SRO) exists in long-range-disordered rocksalt cathodes. We have demonstrated how the SRO controls Li transport through altering local and macroscopic environments. More generally, we observe that electrostatics and ionic sizes strongly affect the SRO in cation-disordered rocksalts of different chemistries. Our findings uncover an important direction for future engineering and optimization of cation-disordered cathode materials to achieve higher capacities and energy densities.

## Methods

### Synthesis

To synthesize LMTO and LMZO, stoichiometric Mn_2_O_3_, TiO_2_, Zr(OH)_4_, and Li_2_CO_3_ (with 5% excess) were dispersed into ethanol and thoroughly mixed using a planetary ball mill (Retsch PM 200) at 300 rpm for 16 h. The mixture was then dried, pelletized, and calcinated at 1100 °C in an argon atmosphere for 2 h, followed by furnace cooling.

### Characterization

The XRD patterns were collected using a Bruker D8 ADVANCE diffractometer equipped with a Cu Kα radiation and an energy dispersive compound silicon strip LYNXEYE XE-T detector in the 2*θ* range of 10–85°. The energy resolution of the detector completely filters out any Fe- or Mn-fluorescence, providing better resolution than a conventional secondary monochromator. The step width and collection time is 0.01° and 2.8 s, respectively. Rietveld refinement was performed using the HighScore Plus software package. Elemental analysis was performed by Luvak Inc. using direct-current plasma emission spectroscopy (ASTM E 1097-12) for the quantitative identification of metal species. The oxygen contents were confirmed using the inert gas fusion method (ASTM E 1019-11). SEM images were obtained on a Zeiss Gemini Ultra-55 analytical field-emission scanning electron microscope. ED patterns were taken after a grain was oriented properly on JEM-2100F using selected area electron diffraction in TEM mode. Time-of-flight (TOF) neutron powder diffraction was performed at room temperature on the Nanoscale Ordered Materials Diffractometer (NOMAD) at the Spallation Neutron Source at Oak Ridge National Laboratory. The samples for the neutron experiment were synthesized using a 7-Li enriched Li_2_CO_3_ source. The PDF patterns were analyzed using the PDFGui software package^43^.

### Electrochemistry

To fabricate electrodes, the product powder was first shaker-milled (SPEX 8000) for 1 h in an argon atmosphere. The milled active material (70 wt%) was then manually mixed with Super C65 carbon black (Timcal, 20 wt%) in a mortar for 30 min, followed by mixing with polytetrafluoroethylene (Dupont, 10 wt%) in an Ar-filled glovebox. The mixture was then rolled into a thin film to be used as the cathode. A coin cell was assembled using 1 M LiPF_6_ (in a volumetric 1:1 mixture of ethylene carbonate and dimethyl carbonate), glass microfiber filters (grade GF/F, Whatman), and Li metal foil as the electrolyte, separator, and anode, respectively. The coin cells were tested on an Arbin battery testing station. PITT measurements were performed on a Solartron Analytical 1470E Celltest System.

### DFT calculations

All the DFT calculations were performed using the Vienna ab Initio simulation package (VASP)^44,45^ with projector-augmented wave^46^ pseudopotentials and the exchange-correlation functional by Perdew, Burke, and Ernzerhof^47^. To correct the DFT self-interaction error, the Hubbard-U correction^48^ was employed for the transition-metal *d* states where needed with values taken from Jain et al.^49^. The U parameters were originally fitted to oxidation enthalpies that have been shown to ensure the robust prediction of relative energies^50^. We employed *k*-point meshes with a reciprocal spacing of 25 *k*-points per Å^−1^ for the Brillouin-zone integration and a plane wave basis set with an energy cutoff of 520 eV. All the DFT energies and atomic forces were converged to 0.001 meV per atom and 20 meV Å^−1^, respectively. Input files for the DFT calculations were generated using the Python Materials Genomics^51^ package.

### Cluster expansion

The cluster expansion (CE) method is used in the present work to enable extensive configurational sampling that would not directly be possible with DFT. The CE method is a lattice model that captures the configurational degrees of freedom (which are the relevant quantity in the present work). CE is based on a formally exact expansion of the formation energy in contributions from different types of interaction between lattice sites. Our CE model was found to reproduce DFT energies with good accuracy (<8 meV per atom) when pair interactions (two lattice sites), triplet interactions (three sites), and quadruplets (four sites) up to the cutoff distances are included in the expansion. The CE model was constructed by fitting to around 500 lattice configurations and their DFT energies. The accuracy was determined by cross-validation, i.e., based on atomic configurations that were not included in the fit. More details are given below.

Cluster expansion Hamiltonians^52^ for each chemical space discussed here were constructed based on the energies of ~500 lattice configurations, where the energies were computed with DFT. Each cluster expansion relied on a basis set of pair interactions up to 7 Å, triplet interactions up to 4.1 Å, and quadruplet interactions up to 4.1 Å, with respect to a rocksalt primitive cell with lattice constant *a* = 3 Å, on top of a baseline of formal-charge electrostatics and a fitted dielectric constant^33^. The effective cluster interactions and dielectric constant were obtained from a L_1_-regularized linear regression fit, with the regularization parameter optimized by cross-validation^53^. The resulting fits yielded an out-of-sample root mean square error of less than 8 meV per atom. All canonical Monte Carlo simulations based on these Hamiltonians were run using the Metropolis-Hastings algorithm.

While cluster expansion models can in principle include the effect of phonon entropy^54,55^, this is rarely done in practice when predicting finite-temperature phase stability as the variation of vibrational entropy with short and long-range order of the cations is much smaller than the effect of configurational entropy^56^. Thus, the effect of lattice dynamics on the cation configuration, and thereby the population of 0-TM, 1-TM, and other Li-TM clusters, can be expected to be negligible compared to the effect of configurational free energy which we sample accurately.

For the connectivity analysis, the definitions of 0-TM, 1-TM, and 2-TM channels of reference^4^ were used. For each composition and temperature, the connectivity was averaged over 600 atomic configurations obtained from MC simulations as previously described. To ensure convergence of the 0-TM Li network size, 3 × 3 × 3 supercells of the MC configurations containing 12,960 cation sites were used.

### Simulation of ED patterns

The LMZO and LMTO atomic configurations equilibrated with Monte Carlo simulations at 1000 °C as described above were used to simulate electron diffraction (ED) images. For this purpose, cubic sections with an edge length of 50 Å (>12,000 atoms) were truncated from the periodic bulk structures defined by 960-atom unit cells. Electron diffraction patterns for the cubic [100] zone axis were computed for each cubic cell using the methods and potentials given by Kirkland^57^, using the potential calculation method described in reference^58^. The final diffraction images were calculated as an incoherent sum of all 500 simulated patterns, and these summed images were smoothed and scaled by amplitude (square root of intensity) to more clearly show the SRO features of the pattern.

## Supplementary information

Supplementary Information

Peer Review File

## Data Availability

The datasets generated and analyzed during the current study are available from the corresponding author on reasonable request.

## References

[1] Olivetti EA, Ceder G, Gaustad GG, Fu X (2017). Lithium-ion battery supply chain considerations: analysis of potential bottlenecks in critical metals. Joule.

[2] Meng YS, Arroyo-de Dompablo ME (2012). Recent advances in first principles computational research of cathode materials for lithium-ion batteries. Acc. Chem. Res..

[3] Lee J (2014). Unlocking the potential of cation-disordered oxides for rechargeable lithium batteries. Science.

[4] Urban A, Lee J, Ceder G (2014). The configurational space of rocksalt-type oxides for high-capacity lithium battery electrodes. Adv. Energy Mater..

[5] Yabuuchi N (2015). High-capacity electrode materials for rechargeable lithium batteries: Li_3_NbO_4_-based system with cation-disordered rocksalt structure. Proc. Natl Acad. Sci. USA.

[6] Yabuuchi N (2016). Origin of stabilization and destabilization in solid-state redox reaction of oxide ions for lithium-ion batteries. Nat. Commun..

[7] Lee J (2015). A new class of high capacity cation-disordered oxides for rechargeable lithium batteries: Li–Ni–Ti–Mo oxides. Energy Environ. Sci..

[8] Freire M (2016). A new active Li–Mn–O compound for high energy density Li-ion batteries. Nat. Mater..

[9] Chen R (2016). Identifying the redox activity of cation-disordered Li–Fe–V–Ti oxide cathodes for Li-ion batteries. Phys. Chem. Chem. Phys..

[10] Chen R (2015). Disordered lithium-rich oxyfluoride as a stable host for enhanced Li^+^ intercalation storage. Adv. Energy Mater..

[11] Takeda N (2017). Reversible Li storage for nanosize cation/anion-disordered rocksalt-type oxyfluorides: LiMoO_2–*x*_LiF (0 ≤ *x* ≤ 2) binary system. J. Power Sources.

[12] Lee J (2017). Mitigating oxygen loss to improve the cycling performance of high capacity cation-disordered cathode materials. Nat. Commun..

[13] House RA (2018). Lithium manganese oxyfluoride as a new cathode material exhibiting oxygen redox. Energy Environ. Sci..

[14] Lee J (2018). Reversible Mn^2+^/Mn^4+^ double redox in lithium-excess cathode materials. Nature.

[15] Van der Ven A, Ceder G (2001). Lithium diffusion mechanisms in layered intercalation compounds. J. Power Sources.

[16] Kang K, Ceder G (2006). Factors that affect Li mobility in layered lithium transition metal oxides. Phys. Rev. B.

[17] Kang K, Meng YS, Bréger J, Grey CP, Ceder G (2006). Electrodes with high power and high capacity for rechargeable lithium batteries. Science.

[18] Rougier A, Gravereau P, Delmas C (1996). Optimization of the composition of the Li_1−*z*_Ni_1+*z*_O_2_ electrode materials: structural, magnetic, and electrochemical studies. J. Electrochem. Soc..

[19] Van der Ven A, Bhattacharya J, Belak AA (2012). Understanding Li diffusion in Li-intercalation compounds. Acc. Chem. Res..

[20] Urban A, Seo DH, Ceder G (2016). Computational understanding of Li-ion batteries. npj Comput. Mater..

[21] Wen CJ, Huggins RA (1981). Chemical diffusion in intermediate phases in the lithium-silicon system. J. Solid State Chem..

[22] Kim JC, Seo DH, Chen H, Ceder G (2015). The effect of antisite disorder and particle size on Li intercalation kinetics in monoclinic LiMnBO_3_. Adv. Energy Mater..

[23] Wen CJ, Boukamp B, Huggins R, Weppner W (1979). Thermodynamic and mass transport properties of “LiAl”. J. Electrochem. Soc..

[24] Kan WH (2018). Unravelling solid-state redox chemistry in Li_1.3_Nb_0.3_Mn_0.4_O_2_ single-crystal cathode material. Chem. Mater..

[25] Cowley JM (1950). X-ray measurement of order in single crystals of Cu_3_Au. J. Appl. Phys..

[26] Reinhard L (1992). Anomalous-X-ray-scattering study of local order in bcc Fe_0.53_Cr_0.47_. Phys. Rev. B.

[27] Mitome M (2004). Transmission electron microscopy and electron diffraction study of the short-range ordering structure of *α*-LiFeO_2_. Acta Crystallogr. Sect. B Struct..

[28] Hauck J (1980). Short-range order and superstructures of ternary oxides AMO_2_, A_2_MO_3_ and A_5_MO_6_ of monovalent A and multivalent M metals related to the NaCl Structure. Acta Crystallogr. Sect. A Cryst..

[29] De Ridder R, Van Dyck D, Van Tendeloo G, Amelinckx S (1977). A cluster model for the transition state and its study by means of electron diffraction. II. Application to some particular systems. Phys. Status Solidi A.

[30] De Ridder R, Van Tendeloo G, Amelinckx S (1976). A cluster model for the transition from the short-range order to the long-range order state in fcc based binary systems and its study by means of electron diffraction. Acta Crystallogr. Sect. A Cryst..

[31] De Fontaine, D. Configurational thermodynamics of solid solutions, in *Solid State Physics* **34**, 73–274 (Academic Press, Cambridge, 1979).

[32] Urban A, Matts I, Abdellahi A, Ceder G (2016). Computational design and preparation of cation-disordered oxides for high-energy-density Li-ion batteries. Adv. Energy Mater..

[33] Richards WD, Dacek ST, Kitchaev DA, Ceder G (2018). Fluorination of lithium-excess transition metal oxide cathode materials. Adv. Energy Mater..

[34] Billinge SJ, Levin I (2007). The problem with determining atomic structure at the nanoscale. Science.

[35] Kikuchi R (1951). A theory of cooperative phenomena. Phys. Rev..

[36] Hodeau JL, Marezio M, Santoro A, Roth RS (1982). Neutron profile refinement of the structures of Li_2_SnO_3_ and Li_2_ZrO_3_. J. Solid State Chem..

[37] Dorrian JF, Newnham RE (1969). Refinement of the structure of Li_2_TiO_3_. Mater. Res. Bull..

[38] Urban A, Abdellahi A, Dacek S, Artrith N, Ceder G (2017). Electronic-structure origin of cation disorder in transition-metal oxides. Phys. Rev. Lett..

[39] Yabuuchi N (2016). Synthesis and electrochemical properties of Li_1.3_Nb_0.3_V_0.4_O_2_ as a positive electrode material for rechargeable lithium batteries. Chem. Commun..

[40] Bréger J (2005). Short-and long-range order in the positive electrode material, Li (NiMn)_0.5_O_2_: a joint X-ray and neutron diffraction, pair distribution function analysis and NMR study. J. Am. Chem. Soc..

[41] Zeng D, Cabana J, Bréger J, Yoon WS, Grey CP (2007). Cation ordering in Li [Ni_*x*_Mn_*x*_Co_(1–2*x*)_]O_2_-layered cathode materials: a nuclear magnetic resonance (NMR), pair distribution function, X-ray absorption spectroscopy, and electrochemical study. Chem. Mater..

[42] Yoon WS (2004). Local structure and cation ordering in O3 lithium nickel manganese oxides with stoichiometry Li[Ni_*x*_Mn_(2−*x*)/3_Li_(1−2*x*)/3_]O_2_ NMR studies and first principles calculations. Electrochem. Solid State.

[43] Farrow C (2007). PDFfit2 and PDFgui: computer programs for studying nanostructure in crystals. ‎J. Phys. Condens. Matter.

[44] Kresse G, Furthmüller J (1996). Efficient iterative schemes for ab initio total-energy calculations using a plane-wave basis set. Phys. Rev. B.

[45] Kresse G, Furthmüller J (1996). Efficiency of ab-initio total energy calculations for metals and semiconductors using a plane-wave basis set. Comput. Mater. Sci..

[46] Blöchl PE (1994). Projector augmented-wave method. Phys. Rev. B.

[47] Perdew JP, Burke K, Ernzerhof M (1996). Generalized gradient approximation made simple. Phys. Rev. Lett..

[48] Dudarev S, Botton G, Savrasov S, Humphreys C, Sutton A (1998). Electron-energy-loss spectra and the structural stability of nickel oxide: an LSDA + U study. Phys. Rev. B.

[49] Jain A (2011). A high-throughput infrastructure for density functional theory calculations. Comput. Mater. Sci..

[50] Wang L, Maxisch T, Ceder G (2006). Oxidation energies of transition metal oxides within the GGA + U framework. Phys. Rev. B.

[51] Ong SP (2013). Python Materials Genomics (pymatgen): a robust, open-source python library for materials analysis. Comput. Mater. Sci..

[52] Sanchez JM, Ducastelle F, Gratias D (1984). Generalized cluster description of multicomponent systems. Phys. A Stat. Mech. Appl..

[53] Nelson LJ, Hart GL, Zhou F, Ozoliņš V (2013). Compressive sensing as a paradigm for building physics models. Phys. Rev. B.

[54] Garbulsky GD, Ceder G (1994). Effect of lattice vibrations on the ordering tendencies in substitutional binary alloys. Phys. Rev. B.

[55] Van De Walle A, Ceder G (2002). The effect of lattice vibrations on substitutional alloy thermodynamics. Rev. Mod. Phys..

[56] Fultz B, Anthony L, Nagel LJ, Nicklow RM, Spooner S (1995). Phonon densities of states and vibrational entropies of ordered and disordered Ni_3_Al. Phys. Rev. B.

[57] Kirkland EJ (2010). Advanced computing in electron microscopy.

[58] Ophus C (2017). A fast image simulation algorithm for scanning transmission electron microscopy. Adv. Struct. Chem. Imaging.

