# Supplementary material for: Hidden structural and chemical order controls lithium transport in cation-disordered oxides for rechargeable batteries
Source: Nat Commun. 2019 Feb 5;10:592. doi: 10.1038/s41467-019-08490-w (PMC6363792; doi:10.1038/s41467-019-08490-w)
Supplement: Supplementary file 1 — Supplementary Information [file 41467_2019_8490_MOESM1_ESM.pdf]

Supplementary Information

Huiwen Ji et al.

Supplementary Figures

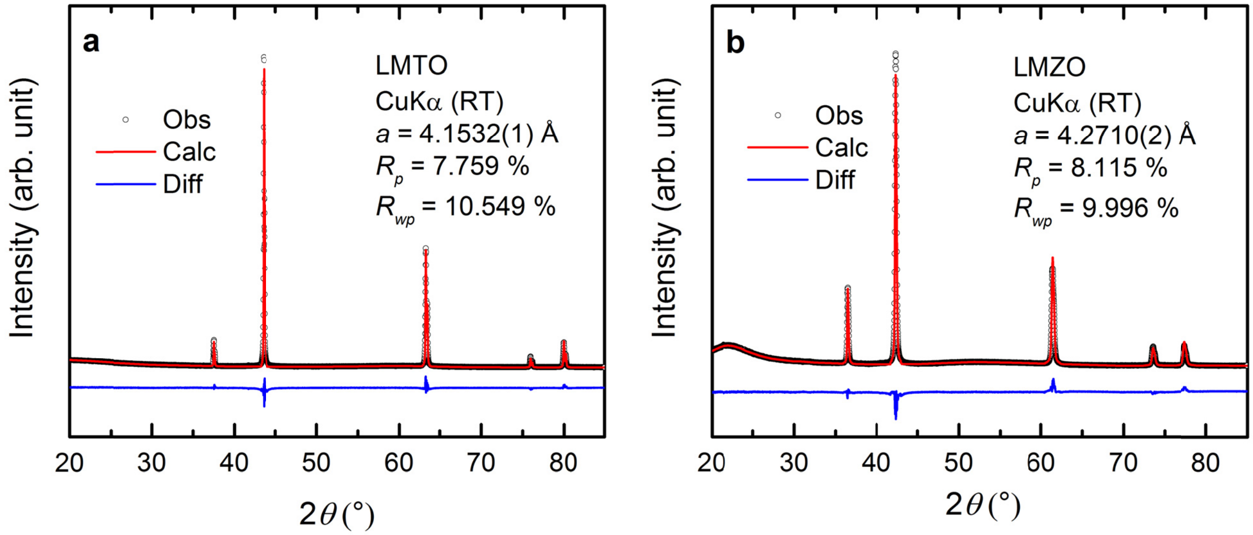

Supplementary Figure 1. Rietveld refinement of XRD patterns of $Li_{1.2}Mn_{0.4}Ti_{0.4}O_2$ (LMTO, **a**) and $Li_{1.2}Mn_{0.4}Zr_{0.4}O_2$ (LMZO, **b**). A rocksalt structural model (space group *Fm*-3*m*) was used for the refinement. The experimental data are plotted as black open circles. The calculated values based on structural models are plotted as solid red lines. The difference between observation and calculation is plotted as solid blue lines. Only the instrumental zero shift, scale factor, lattice constants, and peak-profile parameters *U*, *V*, *W* were refined. The isotropic thermal parameter B_{iso} was fixed at a typical value of 0.5 Å^2. The site occupancies were set to those of the target compositions and were not refined.

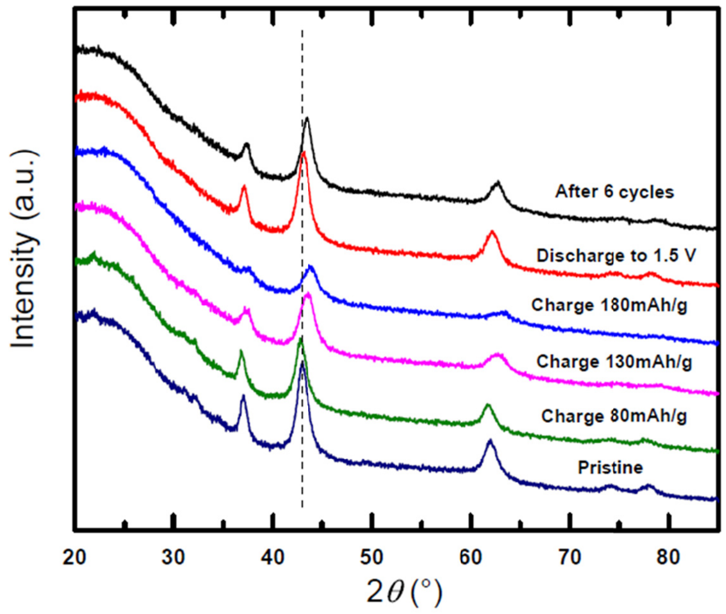

Supplementary Figure 2. Ex-situ XRD patterns of LMZO during the first cycle and after 6 cycles. The cathode films were galvanostatically cycled at 10 mA/g at room temperature. The charging capacity 180 mAh/g roughly corresponds to the extractable capacity with an upper cutoff voltage of 4.7 V. The patterns show a reversible change in lattice parameters, with X-ray peaks recovered to the initial positions of the pristine film. After 6 cycles, the cation-disordered rocksalt structure was retained although the lattice parameter slightly shrank as compared to the pristine sample, indicated by the right-shifted X-ray peaks. The lattice contraction may be associated with lattice densification, which is a common reason that leads to capacity fading in cation-disordered Li-TM oxide cathode materials[1].

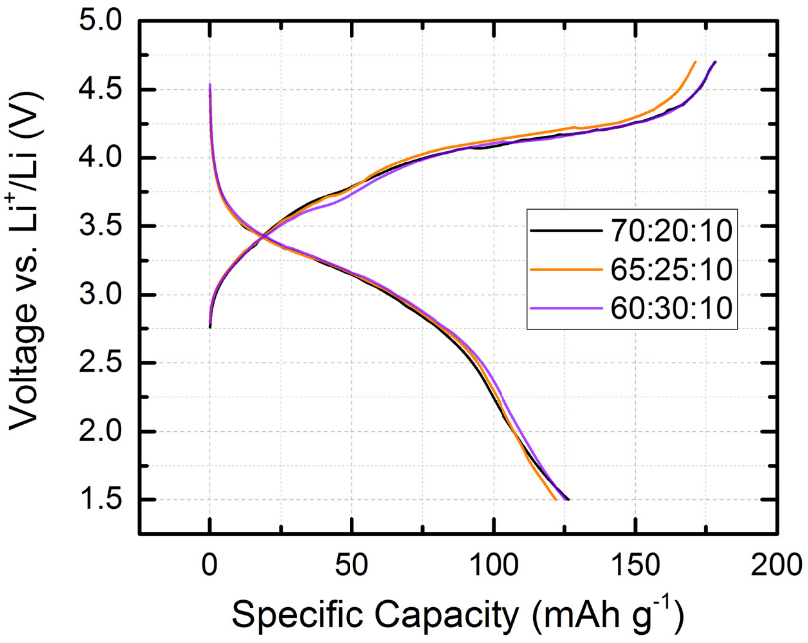

Supplementary Figure 3. First-cycle voltage profiles of LMZO cathode films prepared with various carbon contents. The weight ratios between active material, carbon black and teflon are 70:20:10, 65:25:10 and 60:30:10, respectively.

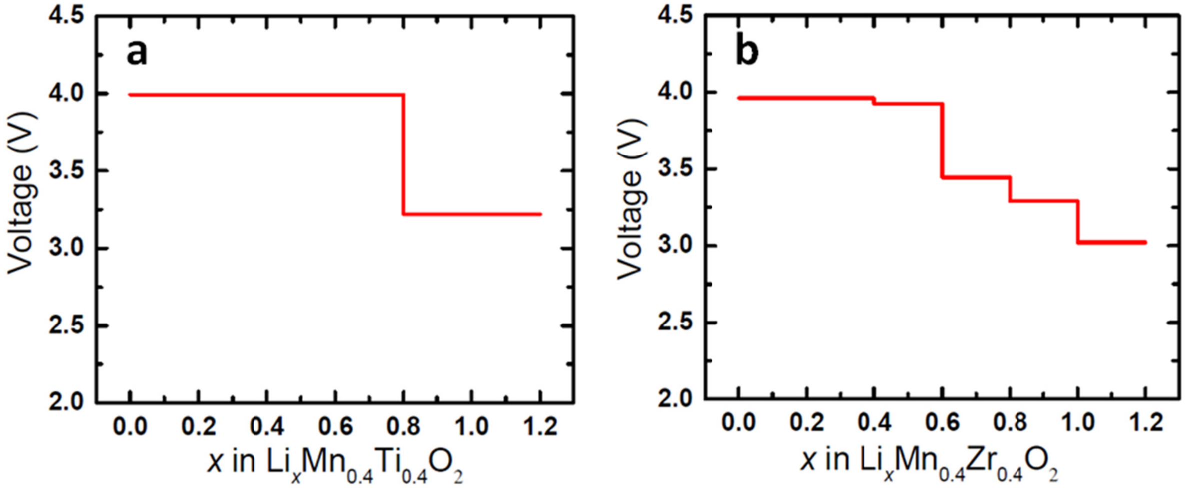

Supplementary Figure 4. Computed voltage profiles of LMTO (**a**) and LMZO (**b**).

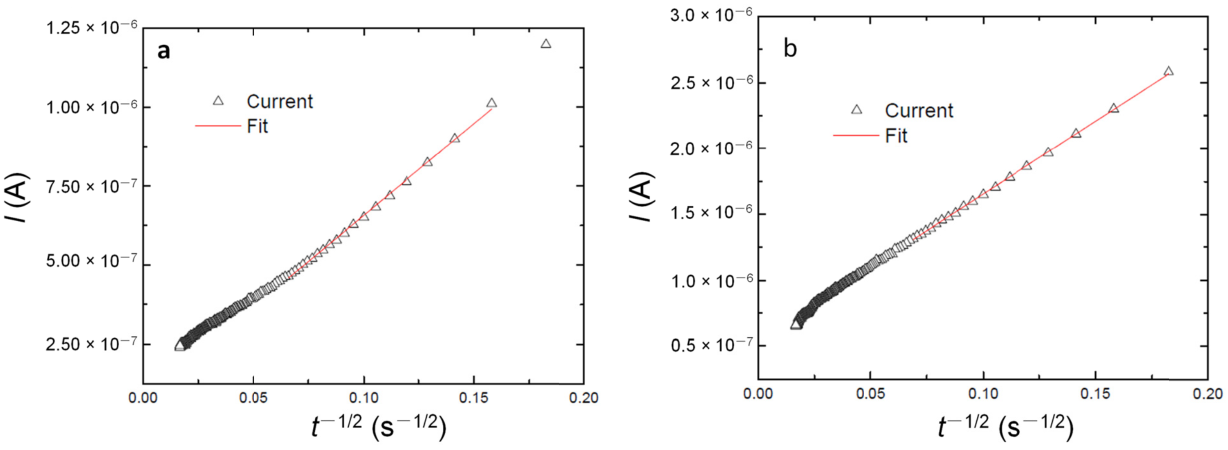

Supplementary Figure 5. Examples of fitting PITT data for LMTO (**a**) and LMZO (**b**). The current–time response was recorded at 2.803 V for LMTO and at 2.604 V for LMZO and is plotted with black triangles. The linear fittings are plotted with solid red lines.

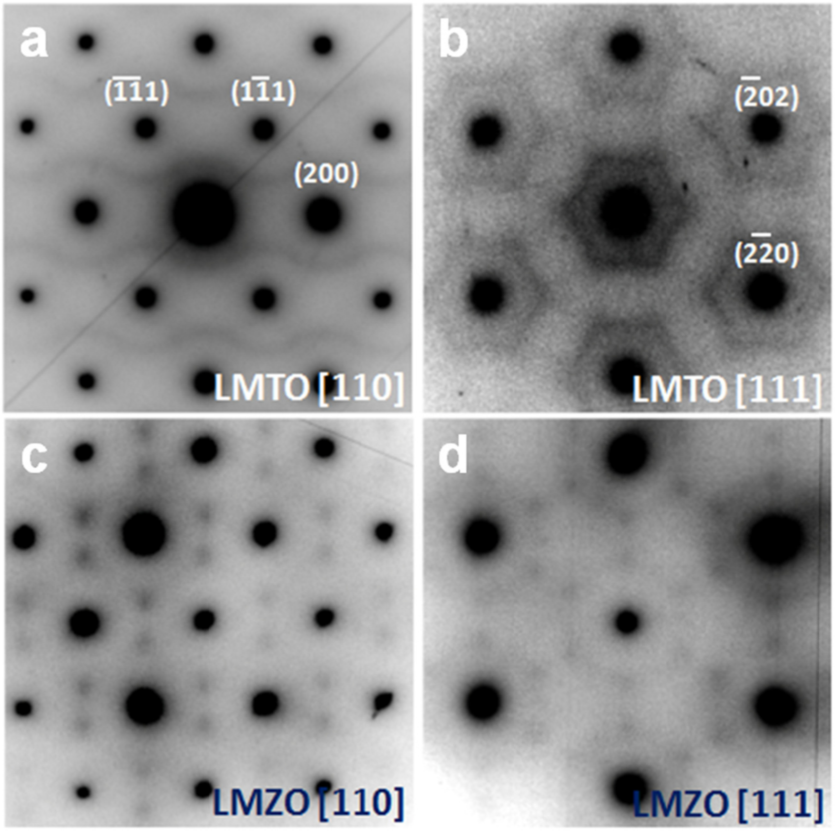

Supplementary Figure 6. ED patterns of LMTO (**a, b**) and LMZO (**c, d**) along the zone axes [110] and [111]. Along each zone axis, the images of LMTO and LMZO are aligned towards the same orientation. The round Bragg spots observed are indexed to the F*m*-3*m* space group, while the diffuse scattering patterns are attributed to SRO. Consistent with the observation along [100] in Figure 3a and 3c, the diffuse scattering patterns are completely different for the two materials, suggesting significant difference in their SRO. In addition, the diffuse scattering patterns of LMZO show intensity maxima that do not exist in the patterns of LMTO, suggesting more pronounced SRO in LMZO.

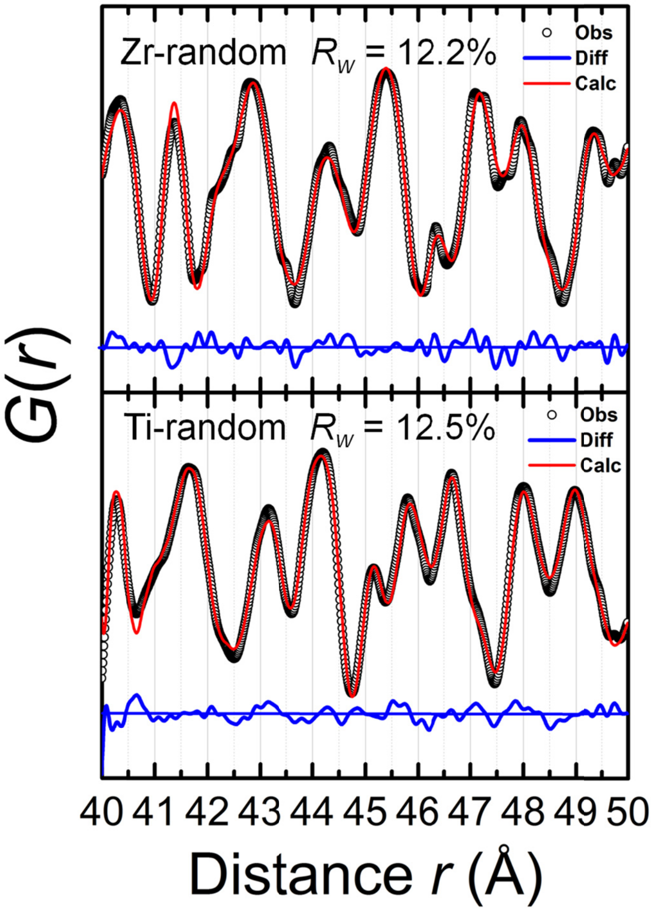

Supplementary Figure 7. Refinement of NPDF data of LMZO (top) and LMTO (bottom) using the random structural model in a long-*r* range. The experimental data are plotted as black open circles. The calculated values based on structural models are plotted as solid red lines. The difference between observation and calculation is plotted as solid blue lines.

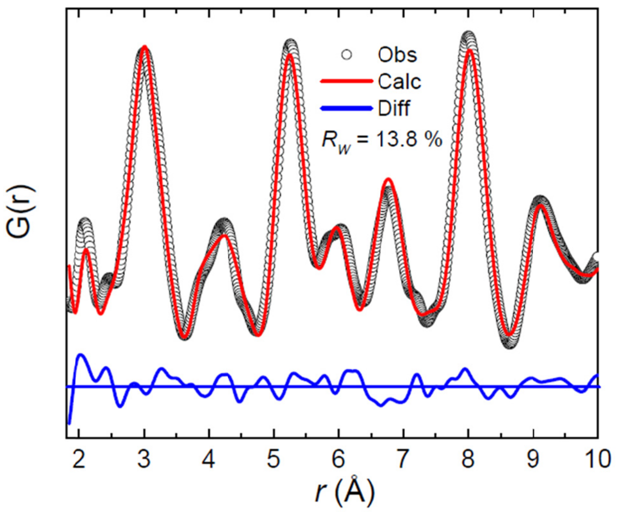

Supplementary Figure 8. An example of NPDF refinement for LMZO using one MC structure within the short-*r* range from 1.8 Å to 10 Å. The experimental data are plotted as black open circles. The calculation based on the MC structural model is plotted as a solid red line. The difference between observation and calculation is plotted as a solid blue line.

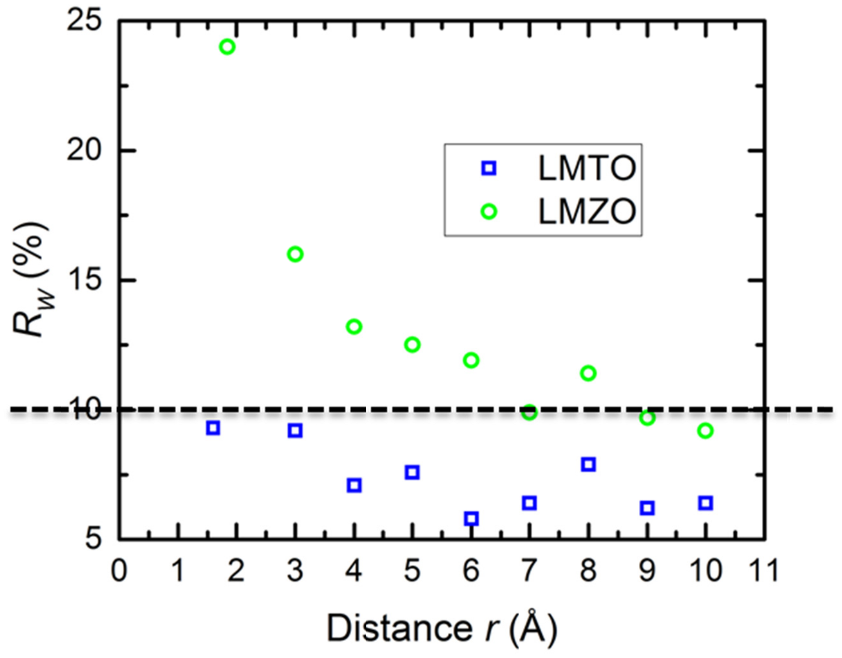

Supplementary Figure 9. R_w as a function of distance r for LMTO and LMZO. The R_w's at the lowest r values for both compounds (1.6 Å for LMTO and 1.85 Å for LMZO) are obtained by refining the NPDF data from the occurrence of the shortest bond to 10 Å, while all other R_w values are obtained by refining the NPDF data within a 10-Å range starting from the r value. For example, the R_w for LMZO at r = 4 Å is obtained by refining the NPDF data from 4 to 14 Å.

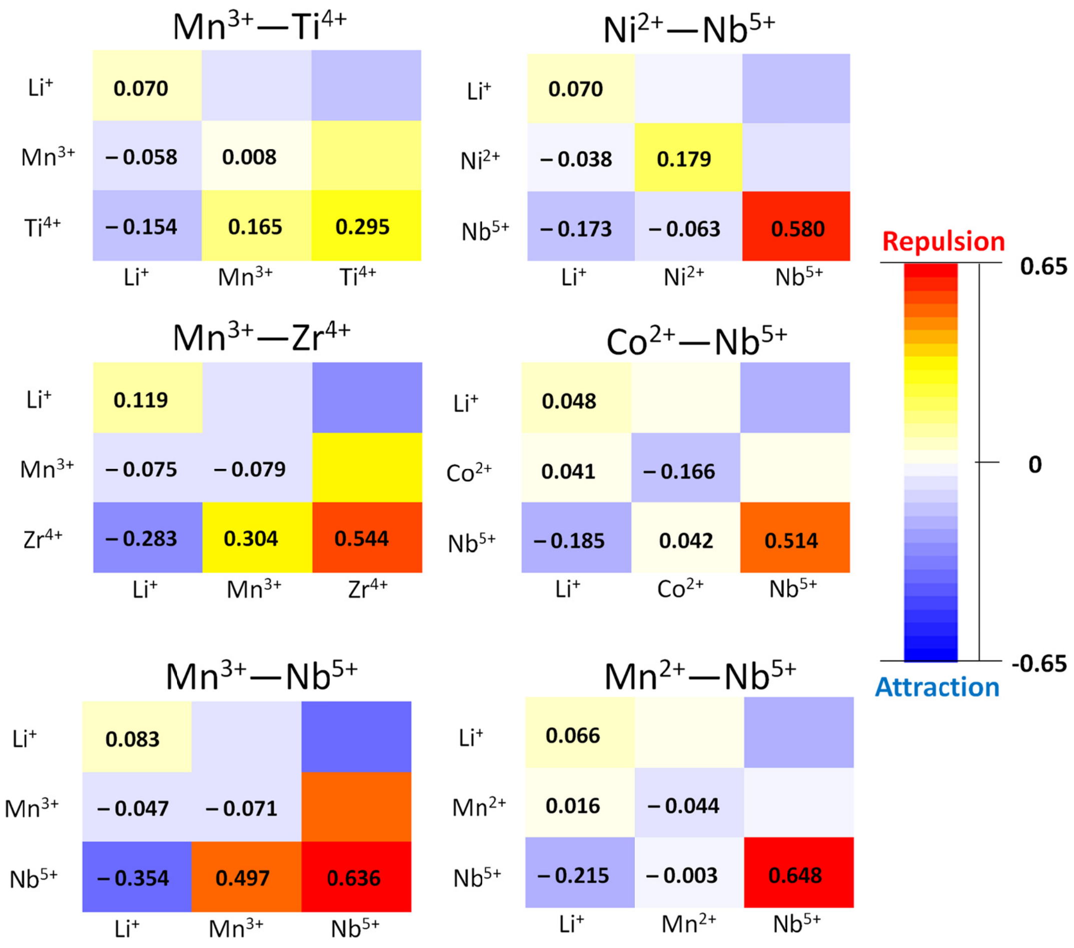

Supplementary Figure 10. Diagrams of Nearest-neighbor pair parameters for various $Li_{1.2}M'_aM''_bO_2$ compounds. The color of a block visualizes the attraction (blue) or repulsion (red) between two metal species.

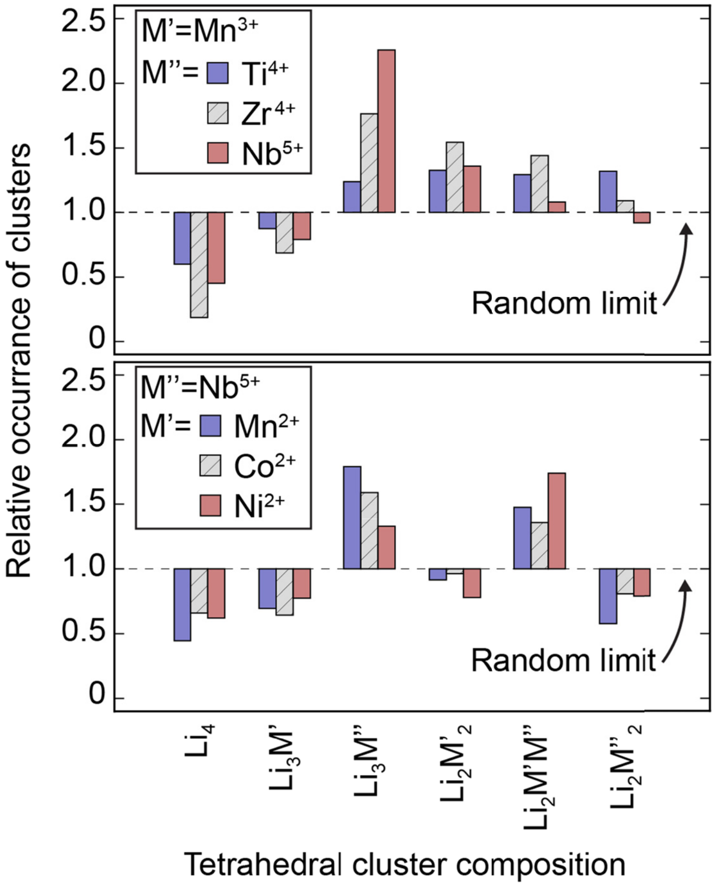

Supplementary Figure 11. Occurrence of tetrahedral clusters in various $Li_{1.2}M'_aM''_bO_2$ compounds relative to the random limit. The stoichiometry of the each $Li_{1.2}M'_aM''_bO_2$ compound is constructed such that charge neutrality is retained.

Supplementary Tables

Supplementary Table 1. Results of elemental analysis by direct-current plasma emission spectroscopy

Materials	Target<br>Li: Mn: Ti: Zr	Measured<br>Li: Mn: Ti: Zr
$Li_{1.2}Mn_{0.4}Ti_{0.4}O_2$	1.2: 0.4: 0.4: 0	1.227: 0.373: 0.399: 0
$Li_{1.2}Mn_{0.4}Zr_{0.4}O_2$	1.2: 0.4: 0: 0.4	1.218: 0.412: 0: 0.370

Supplementary Table 2. Refinement results of NPDF data for LMTO and LMZO in a short-*r* range between the occurrence of the shortest M-O bond (1.6 Å for LMTO and 1.85 Å for LMZO) and 10 Å using the random structural model

	LMTO (short-*r* range)	LMZO (short-*r* range)
Space Group	*Fm*-3*m*	*Fm*-3*m*
a (Å)	4.1674	4.2837
δ_1*	0.26213	1.6
U_{iso} (Å^2)	0.019497	0.03134
R_W	9.3%	24.0%

Supplementary Table 3. Refinement results of NPDF data for LMTO and LMZO in a long-*r* range between 40 Å and 50 Å using the random structural model

	LMTO (long-*r* range)	LMZO (long-*r* range)
Space Group	*Fm*-3*m*	*Fm*-3*m*
a (Å)	4.1582	4.2740
$\delta_1{}^*$	0.26213	1.6
U_{iso} (Å^2)	0.014208	0.0186
R_W	12.5%	12.2%

*δ_1 is a high-temperature vibrational correlation parameter and modifies peak widths at short-*r* values. Therefore, δ_1 was only refined within the short-*r* range. The obtained value was then used for the long-*r* range without further refinement.

Supplementary Table 4. Refinement results of NPDF data for LMZO within the short-*r* range (from 1.8 Å to 10 Å) using one MC structure

	LMZO (short-*r* range)
Space Group	*P*1
a (Å)	9.11118
b (Å)	10.8747
c (Å)	13.3222
α	68.2145
β	81.5379
γ	74.7896
δ_1	1.54324
U_{iso} (Å^2)	0.009486
R_W	13.8%

Supplementary Table 5. Occurrence of various tetrahedral clusters in LMTO and LMZO*

**Cluster composition**	**$Li_{1.2}Mn_{0.4}Ti_{0.4}O_2$ 1000 °C**	**$Li_{1.2}Mn_{0.4}Zr_{0.4}O_2$ 1000 °C**	**$Li_{1.2}Mn_{0.4}M'_{0.4}O_2$, Random limit**
**Tetrahedral clusters**			
Li_4	0.076	0.024	0.127
Li_3M	0.367	0.426	0.348
Li_3Mn	0.152	0.119	0.174
Li_3M'	0.215	0.307	0.174
Li_2M_2	0.454	0.479	0.348
Li_2Mn_2	0.115	0.135	0.087
Li_2MnM'	0.224	0.250	0.174
$Li_2M'_2$	0.115	0.095	0.087

* Only Li-rich tetrahedral clusters relevant to Li migration (0-TM, 1-TM, 2-TM) are listed.

Supplementary Notes

Supplementary Note 1: Elemental analysis. The compositions of the synthesized compounds were verified to be nearly identical to the target compositions using direct-current plasma emission spectroscopy.

Supplementary Note 2: Ex-situ XRD of LMZO. Because LMZO is a new compound first reported in this work, we performed ex-situ XRD on the cycled products to determine its stability over cycling.

Supplementary Note 3: Computed voltage profiles of LMTO and LMZO. The voltage curves were computed by first enumerating a large number of possible configurations for a given cathode material at various Li levels. After collecting the ground state configurations, the voltage profiles were then computed using the battery builder function from pymatgen.[2] The computed voltage curves indicate that both materials are predicted to operate in a similar voltage window despite their slightly different voltage profiles. Therefore, the performance of LMZO is unlikely limited by thermodynamics. The open-circuit voltage of LMZO is calculated to be ~ 0.2 V lower than that of LMTO, consistent with the observation from electrochemical tests.

Supplementary Note 4: Li chemical diffusivity analysis. The PITT tests were performed on LMTO and LMZO. Both materials were charged from the open-circuit voltages to 4.7 V with a 0.01-V incremental step interval. At each step, the voltage was held constant for 1 h, and the corresponding current–time response was recorded. To determine the Li chemical diffusivity, an equation developed by Wen, Boukamp, and Huggins[3] based on Fick's second law was used. The equation was applied in the short-time approximation as follows:

$$I(t) = \frac{QD^{1/2}}{L\pi^{1/2}}\frac{1}{\sqrt{t}} \qquad t \ll \frac{L^2}{D} \qquad (1)$$

In Supplementary Equation (1), *I* is the current; *D* is the Li chemical diffusivity; and *L* represents the diffusion length, which in this case is half of the average particle size, 50 nm. Within the short-time region (typically within 400 s), a plot of *I* as a function of $t^{-1/2}$ should give a linear response. The Li diffusivity *D* can be extracted from the slope of this linear region. Theoretically, both the short-time and long-time approximations should give the same results. However, the long-time approximation condition was never fulfilled within the limited time frame of this experiment and with the slow kinetics of the materials.

The slope from fitting *I* vs. $t^{-1/2}$ for LMTO in Supplementary Figure 5a is 5.810×10^{-6} C·s$^{-1/2}$. The accumulated charge for this voltage step is 0.001162 C, and the diffusion length *L* is 50 nm. Therefore, the Li chemical diffusivity is 1.963×10^{-15} cm^2/s for this step. Likewise, the slope from fitting *I* vs. $t^{-1/2}$ for LMZO in Supplementary Figure 5b is 1.094×10^{-5} C·s$^{-1/2}$. The accumulated charge is 0.003174 C. The resulting Li diffusivity is 9.324×10^{-16} cm^2/s.

Supplementary Note 5: Dimension analysis of short-range order. The dimension of short-range order in $Li_{1.2}Mn_{0.4}Ti_{0.4}O_2$ and $Li_{1.2}Mn_{0.4}Zr_{0.4}O_2$ is estimated through refining NPDF data using random structural models in various distance ranges, as shown in Supplementary Figure 9. For $Li_{1.2}Mn_{0.4}Ti_{0.4}O_2$, we observe that in a distance window from 4 to 14 Å the R_w for the refinement already drops below 5%, suggesting that the length scale of short-range order is less than one unit cell. In contrast, for $Li_{1.2}Mn_{0.4}Zr_{0.4}O_2$, the R_w plateaus at around 10 % beyond 8 Å, indicating that the impact of short-range order extends to ~2 unit cells in $Li_{1.2}Mn_{0.4}Zr_{0.4}O_2$ and is thus longer-ranged than in $Li_{1.2}Mn_{0.4}Ti_{0.4}O_2$.

Supplementary Methods

Supplementary Method 1: Refinement of NPDF data within the short-*r* range using MC-equilibrated structures. For each composition, 10 atomic configurations (each with 60 cations) were first obtained from MC equilibration at 1273 K. The atomic coordinates and lattice parameters of all the structures were relaxed using DFT to capture local variations in bond lengths. Each MC structure was then individually fit to the experimental pattern of the corresponding composition. A typical refinement for LMZO using one MC structure is shown in Supplementary Figure 8. The MC structure does not have any symmetry and is in Space Group *P*1. The lattice constants *a*, *b*, *c*, angles α, β, γ, vibrational correlation parameter δ_1, and isotropic thermal parameter U_{iso} were refined, and the results are presented in Supplementary Table 4. After individual fitting, we numerically averaged the calculated profiles based on the 10 configurations and produced a one-dimensional PDF *G*(*r*), which was eventually compared with the experimental *G*(*r*), as shown in Figure 3f and 3h in the main manuscript.

Supplementary Method 2: Details about connectivity analysis. The connectivity analysis is performed on MC structures equilibrated at 1000°C. Each MC structure contains 480 cation sites, of which 288 are decorated with Li ions. The connectivity function is defined as following:

$$P(n) = Pr\left\{\sum_{j=1}^{N} I\left(u_j; n\right) = 1\right\}/N \times 100 \qquad (2)$$

where $I\left(u_j; n\right)$ is an indicator to record whether Li ion u_j is in a Li network of at least n Li sites. If the Li ion u_j is in a Li network of at least n Li sites, then $I\left(u_j; n\right) = 1$; otherwise $I\left(u_j; n\right) = 0$. N is the total number of Li sites in a MC structure. The resulting $P(n)$'s are averaged over the

600 sampled MC structures for each composition to obtain the connectivity plots shown in Figure 4(b).

Supplementary Method 3: Nearest-neighbor pair (NNP) parameters for effective interaction between cation species. We present the nearest-neighbor pair (NNP) parameters for six representative compositions ($Li_{1.2}Mn_{0.4}Ti_{0.4}O_2$, $Li_{1.2}Mn_{0.4}Zr_{0.4}O_2$, $Li_{1.2}Mn_{0.6}Nb_{0.2}O_2$, $Li_{1.2}Ni_{0.4}Nb_{0.4}O_2$, $Li_{1.2}Co_{0.4}Nb_{0.4}O_2$, and $Li_{1.2}Mn_{0.4}Nb_{0.4}O_2$) in Supplementary Figure 10. By comparing the occurrence of a cation pair in the first neighboring shell in MC structures to that in a random structure, net attraction (negative NNPs) or repulsion (positive NNPs) can be extracted between the two cation species.

For each composition, 600 atomic configurations sampled from MC simulations as previously described were analyzed and averaged to obtain the reported nearest-neighbor statistics. The NNP parameter is defined as following:

$$\mathrm{NNP}(\mathrm{A}-\mathrm{B}) = 1 - \frac{\mathrm{n_{MC}(B)}}{\mathrm{n_{random}(B)}}, \quad (3)$$

where A and B are two types of cations; $\mathrm{n_{MC}(B)}$ is the number of cations of type B within the first neighboring shell around cation A averaged over 600 MC structures; and $\mathrm{n_{random}(B)}$ is the number of cations of type B within the first neighboring shell surrounding cation A based on a random model. If NNP(A–B) < 0, the effective interaction between A and B ions is attractive; if NNP(A–B) > 0, the interaction is repulsive; and if NNP(A–B) = 0, the interaction vanishes. For example, if we consider the pair of Li^+ and Ti^{4+} in LMTO, the statistical number of Ti^{4+} ions within the first neighboring shell of Li^+ is $12 \times 0.2 = 2.4$, whereas the average number of Ti^{4+} ions

counted from the MC structures is 2.769. Therefore, NNP(Li^{+}–Ti^{4+}) is –0.154, indicating net attractive interaction between the two species.

Supplementary References

1. Lee, J. *et al.* A new class of high capacity cation-disordered oxides for rechargeable lithium batteries: Li–Ni–Ti–Mo oxides. *Energ. Environ. Sci.* **8**, 3255–3265 (2015).
2. Ong, S. P. *et al.* Python Materials Genomics (pymatgen): A robust, open-source python library for materials analysis. *Comput. Mater. Sci.* **68**, 314–319 (2013).
3. Wen, C. J., Boukamp, B., Huggins, R. & Weppner, W. Thermodynamic and mass transport properties of “LiAl”. *J. Electrochem. Soc.* **126**, 2258–2266 (1979).
